# Comparing two-sample log-linear exposure estimation with Bayesian model-informed precision dosing of tobramycin in adult patients with cystic fibrosis

**DOI:** 10.1128/aac.01040-24

**Published:** 2025-01-10

**Authors:** Dominic M. H. Tong, Maria-Stephanie A. Hughes, Jasmine Hu, Jeffrey C. Pearson, David W. Kubiak, Brandon W. Dionne, Jasmine H. Hughes

**Affiliations:** 1 InsightRX 558247 , San Francisco, California, USA; 2 Department of Pharmacy, Brigham and Women's Hospital 1861 , Boston, Massachusetts, USA; 3 School of Pharmacy and Pharmaceutical Sciences, Northeastern University 1848 , Boston, Massachusetts, USA; Providence Portland Medical Center, Portland, Oregon, USA

**Keywords:** tobramycin, cystic fibrosis, population pharmacokinetic model, Bayesian estimation, two-sample log-linear regression

## Abstract

Tobramycin dosing in patients with cystic fibrosis (CF) is challenged by its high pharmacokinetic (PK) variability and narrow therapeutic window. Doses are typically individualized using two-sample log-linear regression (LLR) to quantify the area under the concentration-time curve (AUC). Bayesian model-informed precision dosing (MIPD) may allow dose individualization with fewer samples; however, the relative performance of these methods is unknown. This single-center retrospective analysis included adult patients with CF receiving tobramycin from 2015 to 2022. Tobramycin concentrations were predicted using LLR or Bayesian estimation with two population PK models (Hennig and Alghanem). Then, both methods were used to estimate the AUC for simulated patients. For Bayesian estimation, AUC estimation with flattened priors and limited sampling strategies were also assessed. Predictions were evaluated using normalized root mean square error (nRMSE), mean percent error (MPE), and accuracy. The data set included 70 treatment courses, with 32 not evaluable by LLR due to detection limits or timing issues. Bayesian estimation demonstrated worse accuracy (47.1%–50.7% vs 75.7%), higher MPE (24.2%–32.4% vs −2.4%), and higher nRMSE (35.0%–39.4% vs 24.8%) than LLR for peak concentrations but performed better on troughs (accuracy: 92.0%–92.9% vs 84.6%). Bayesian estimation with flattened priors and a single sample at 4 h was comparable to LLR performance, with better accuracy (42.9%–68.0% vs 41.1% LLR), comparable MPE (−2.3% to −3.7% vs −0.5%) and nRMSE (11.3%–21.6% vs 17.3%). Bayesian estimation with one concentration and flattened priors can match LLR prediction accuracy. However, popPK models must be improved to better estimate peak samples.

## INTRODUCTION

Cystic fibrosis (CF) affects over 70,000 people as the most common life-threatening genetic disorder (1, 2). Managing CF is difficult due to frequent pulmonary exacerbations often caused by *Pseudomonas aeruginosa*, which can be treated with intravenous tobramycin in combination with other antipseudomonal antimicrobials. Tobramycin dosing is challenging due to its narrow therapeutic range, balancing bactericidal activity against risks of nephrotoxicity and ototoxicity. Due to high inter-individual variability in tobramycin pharmacokinetics (PK), therapeutic drug monitoring (TDM) is routinely performed (1, 3). The primary exposure targets are an area under the concentration-time curve (AUC) to minimum inhibitory concentration (MIC) of 80–110 mg h/L over 24 h for an MIC of 1 (4–7), and a peak concentration of 20–40 mg/L (8, 9).

Tobramycin exposure is often estimated using the two-sample log-linear regression (LLR) method, which uses two serum concentrations to calculate an AUC:MIC. While these calculations can be performed manually, this approach comes with significant limitations. LLR requires two well-timed samples from a single dosing interval, imposing logistical challenges on hospital staff. It also only uses samples from this one dosing interval, meaning that AUC estimation does not become more accurate as additional patient data becomes available. LLR also ignores all previously known information about the drug, including estimates of pharmacokinetic parameters like clearance and volume of distribution from previous studies.

An alternative approach is Bayesian model-informed precision dosing (MIPD), which leverages prior knowledge from population pharmacokinetic (popPK) models and patient TDM samples to estimate tobramycin exposure. This approach can reduce the operational burden on hospital staff by requiring fewer TDM samples and offering flexibility in sampling time (10). While LLR assumes one-compartment pharmacokinetics, popPK models permit multiple distribution compartments, which may better reflect tobramycin pharmacokinetics (11). The use of popPK models also means covariate effects can be used to predict patient exposures even before any TDM samples are drawn, allowing doses to be tailored to the patient earlier.

Bayesian estimation also accounts for measurement and other residual error, making it more robust to measurement errors than the LLR method. LLR assumes that assay error is a constant percentage of the measured concentration, which greatly overestimates the credibility of low serum concentrations compared to higher ones (12). Bayesian approaches can also allow use of below limit of quantification (LOQ) samples (13) instead of discarding them like in LLR. However, because Bayesian estimation balances model priors with patient data, Bayesian estimation adapts slower to patients with atypical PK parameters compared to LLR. One approach for reducing the impact of this model misspecification is to use flattened priors (14, 15), where the weight of the model prior is reduced relative to the data.

Here, we evaluate Bayesian estimation using two popPK models and the two-sample LLR method on their ability to predict measured tobramycin concentrations and estimate a simulated AUC. We use a typical clinical scenario with two concentrations per dosing interval as well as a limited sampling scenario, to assess the advantages and limitations of Bayesian MIPD for personalized tobramycin dosing in patients with CF.

## MATERIALS AND METHODS

### Patient data collection

Routine clinical care data of adult patients with CF treated with intravenous tobramycin at Brigham and Women’s Hospital between June 2015 and July 2022 were entered into the InsightRX Nova Bayesian MIPD platform. These data included dosing information (dose administration times, amounts, and infusion rate), patient demographic data (height, weight, age, and sex), and patient lab data (value and timing of serum creatinine and tobramycin serum concentrations). The tobramycin assay used was linear over a range of 0.4–10 mg/L, with a LOQ of 0.4 mg/L. Assay coefficient of variation (CV) was 2% for a mean sample of 8.6 mg/L and 3.9% for a mean sample of 2.3 mg/L. Patients were included in this analysis if they were at least 18 years of age, had at least two doses of tobramycin, and at least two concentrations drawn during treatment. Treatment courses were excluded if they had a tobramycin concentration drawn during infusion or were identified as outliers with an absolute value of conditional weighted residual (CWRES) of more than five after fitting with the Hennig popPK model.

### Population pharmacokinetic model literature review

A literature search was conducted to identify population pharmacokinetic models that described tobramycin PK in adults with or without CF. Models were included in this analysis if they were (i) developed on a population that closely aligned with the data set analyzed here (i.e., an adult population with CF); (ii) used routinely collected covariates; (iii) developed in NONMEM; (iv) not superseded by a newer model fit; and (v) developed after the year 2000. The last criteria derive from our experience in implementing and evaluating over 200 popPK models from the literature, from the estimation methods being more approximate before 2000 (16), and from the lack of modern consensus on what constitutes proper model development and diagnostics in models developed before 2000 (17, 18).

### Two-sample LLR and population pharmacokinetic model predictive performance

**Fig 1:**
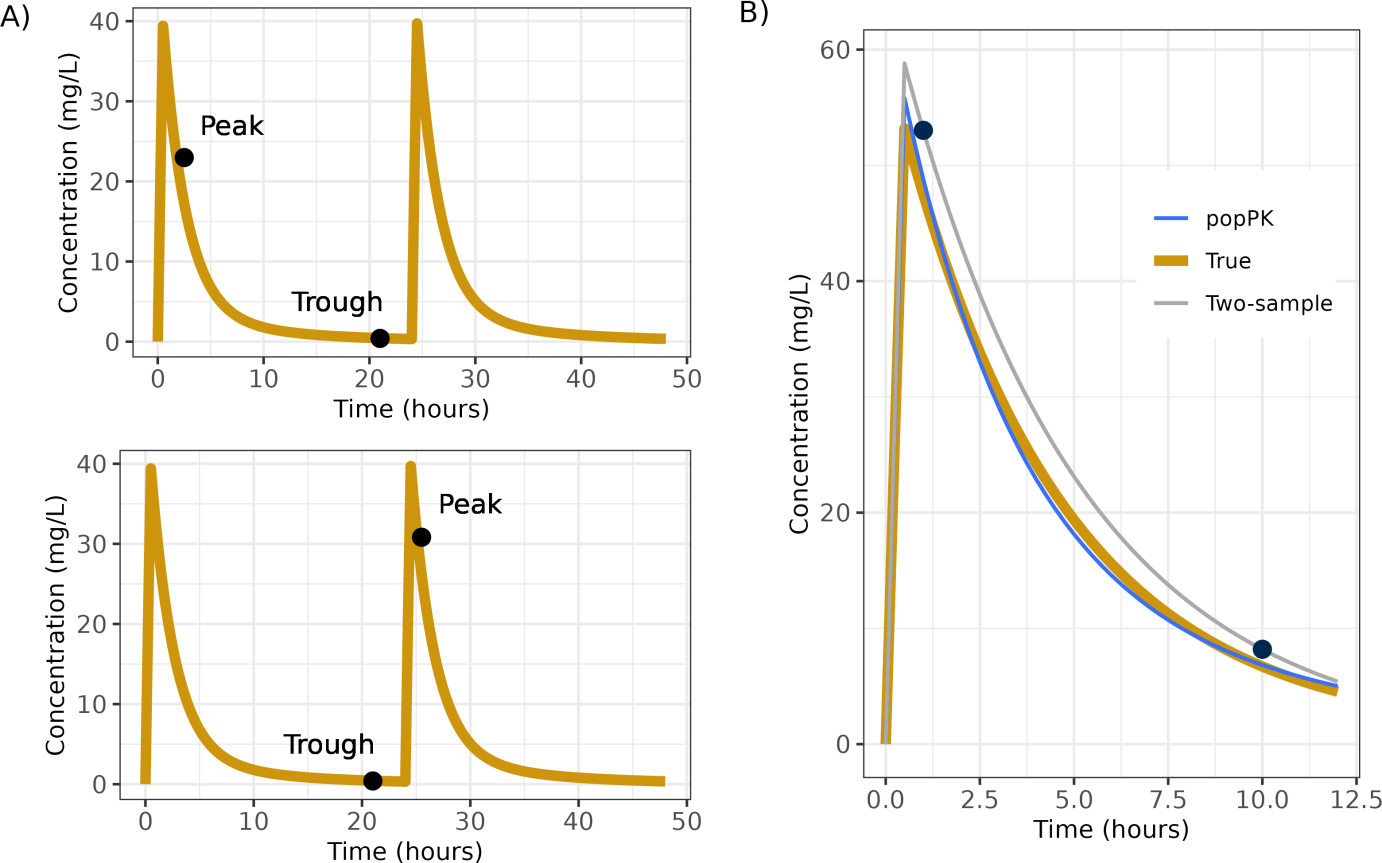
(**A**) Tobramycin sample groups. Peaks and troughs taken within the same dosing interval were grouped. Troughs and peaks taken with exactly one dose between were also grouped. (**B**) Using two-sample LLR and Bayesian estimation methods to estimate concentrations or AUC. In this example, a popPK model was used to simulate patient PK (gold line, “True”) and two simulated tobramycin concentrations (closed circles) were drawn with measurement error. PK parameters were estimated using either the two-sample LLR (gray) or Bayesian estimation method (blue) and used to simulate the concentration-time curve.


Two-sample LLR requires two samples within the same dosing interval, so we defined a group of concentrations (i.e., peak-trough pairs, as defined in Fig. 1A) to be a peak and a trough within one dosing interval or a trough followed by a dose and a peak sample. Peak tobramycin concentrations were defined as concentrations taken within 3 h of the end of the tobramycin infusion; trough tobramycin concentrations were defined as concentrations taken 4+ hours after the end of the infusion. Concentrations that did not meet definitions for peak or trough, or that were below LOQ, were discarded in LLR analysis. LLR was performed according to the clinical standard at Brigham and Women’s Hospital. Two drug concentrations *C*_1_ and *C*_2_, taken at times *t*_1_ and *t*_2_ after a dose *D* with infusion length $t_{inf}$, and dosing interval $t_{interval}$, were used to estimate a one-compartment PK model with elimination coefficient *k*, volume of distribution $V_{d}$, and clearance CL as follows:

$$\begin{array}{l} k=\frac{ln\left(\frac{c_{1}}{c_{2}}\right)}{t_{1}-t_{2}}\\ V_{d}=\frac{D\times \left(1-e^{-k\cdot t_{inf}}\right)\times e^{-k\left(t_{1}-t_{inf}\right)}}{t_{inf}\;\times k\times c_{1}\times \left(1-e^{-k\cdot t_{interval}}\right)}\\ CL=k\cdot V_{d} \end{array}$$

Samples below the limit of quantification (LOQ) of 0.4 mg/L were discarded in LLR.

The predictive performance of popPK models was assessed iteratively using Perl-speaks-NONMEM version 5.2.6 (19). Population PK parameters plus patient covariates were used to predict the first group of concentrations; subsequent groups of concentrations were predicted using individual PK parameters estimated using maximum *a posteriori* Bayesian estimation informed by all previously known concentrations. Concentrations below the LOQ were handled using the likelihood (or M3) method (13, 20) for Bayesian estimation, where the likelihood for an observation below LOQ was defined to be the likelihood of that observation being below the LOQ given the model (as opposed to how the likelihood is defined for non-censored samples, i.e., as a function of the difference between observed and predicted concentration).

Figure 1B illustrates the differences between the LLR and Bayesian methods. It shows a simulated true tobramycin concentration curve (gold), with a peak and a trough TDM sample taken (black). Because these samples include simulated measurement error, they do not fall exactly on the true concentration curve. The LLR method fits a one-compartment model directly to the samples. The two-compartment popPK model fits a concentration curve to the sample data while allowing for residual variability.

### Area under the concentration-time curve simulation and estimation

As *true* AUC estimation requires dense sampling, which is not available with real-world data, the AUC estimation performance was assessed using simulation. Concentration-time curves were simulated using PK models following a dose of 10 mg/kg infused over 30 min, and the “true” AUC was calculated by integrating under the curve. In the first experiment comparing AUC estimation using LLR and popPK models using different flattened priors weights, samples were extracted at 1 and 10 h after dose from the simulated curves with residual variability applied. In the second experiment, the LLR results using samples at 1 and 10 h were compared to one sample at various times using popPK models to estimate AUC. The model different from the simulation model (“mis-matched model”) was emphasized as there is always some model misspecification in clinical practice. Simulated patients used the same covariates as the real patients (age, weight, height, and sex), but with inter-individual variability in PK parameters determined by randomly drawing five times per patient from the distributions described in the original publications to generate five separate individuals. Simulation and AUC estimation were performed in R version 4.1.0 (21), using PK models implemented via PKPDsim (22).

### Statistics and error metrics

The difference between predicted values (pred_i_) and measured or simulated true values (obs_i_) (including AUCs, peaks, and troughs) was described using normalized root mean square error (nRMSE) and mean percent error (MPE), as defined below:


$$\begin{array}{l} RMSE=\sqrt{\frac{\sum_{i=1}^{N}{\left({pred}_{i}-{obs}_{i}\right)}^{2}}{N}}\\ nRMSE=\frac{RMSE}{\frac{1}{N}\sum_{i=1}^{N}{obs}_{i}}\\ MPE=\frac{1}{N}\sum_{i=1}^{N}\frac{{pred}_{i}-{obs}_{i}}{{obs}_{i}}\;\times \;100\% \end{array}$$

Sample variability in these metrics was assessed across 1,000 bootstrapped samples of all patients in the data set. The overlap of the 2.5th–97.5th percentile of these bootstrapped metrics was compared to assess statistical significance. Statistical analysis was performed in R (21).

The clinical relevance of differences in methods was assessed using “accuracy,” defined as the ability to produce estimates within a tolerable degree of error (23, 24). Tolerable error ranges were defined per exposure metric: peak concentration predictions were considered accurate if the prediction was within 20% of the measured value; trough predictions if the predicted and measured value were both above 1 mg/L or below 1 mg/L; and AUC estimates were considered accurate if the predicted AUC was within 20% of the simulated true value.

## RESULTS

### Patients and data collection

The final data set included 50 adult patients with CF across 70 treatment courses between June 2015 and July 2022, summarized in Table 1. These patients provided 345 tobramycin concentrations, with a median peak sample time of 1.6 h after the end of infusion and median trough sample time of 19.8 h after the end of infusion. Sixteen treatment courses were removed for having a tobramycin concentration during infusion and six were removed as outliers with an absolute CWRES over five after fitting with the Hennig model.

**TABLE 1:** Patient characteristics of the model development populations of Hennig and Alghanem and this study population^*a*^

Characteristic (unit)	Hennig (adults only)	Alghanem	This study
No. of patients	208	166	50
No. of CF patients	114	166	50
No. of treatment courses	N/A	1,075	70
No. of tobramycin concentrations	2,055	2,238	345 (134 peaks, 206 troughs, 5 other)
No. of concentrations below limit of quantification	~95^*b*^	5	86
Sex (male/female)	99/109	81/85	28/22
Age (years)	31.7 (18.0–85.0)	23 (14–66)	32.7 (22.2–58.8)
Baseline serum creatinine (mg/dL)	0.77 (0.43–4.0)	0.80 (0.33–2.30)	0.73 (0.38–1.31)
Weight (kg)	58.0 (37.0–120.0)	50 (30–86)	58.2 (39–90.9)
Height (cm)	167.0 (148.4–190.0)	163 (139–191)	165.1 (149.9–182.9)
Creatinine clearance (mL/min)	71.5 (22.8–144.6)	93 (33.8–144.6)	113.1 (25.4–251.3)

^*a*^ Values are count or median (range) for the Hennig and Alghanem models or median (5th–-95th percentile) for this study. N/A, not provided.

^*b*^ Hennig gives a percentage of all concentrations below limit of quantification for adults and children in their patient population; we use this proportion to estimate the number of concentrations BLOQ for adults.

### Population pharmacokinetic model and LLR method predictive performance

The popPK models from Hennig (25) and Alghanem (26) for tobramycin in adult CF patients were identified from our literature search and their development populations are summarized in Table 1. Both models have two compartments, which is consistent with known tobramycin kinetics (11), and showed reasonable predictive performance using standard popPK diagnostics (Fig. S1 and S2).

**Fig 2:**
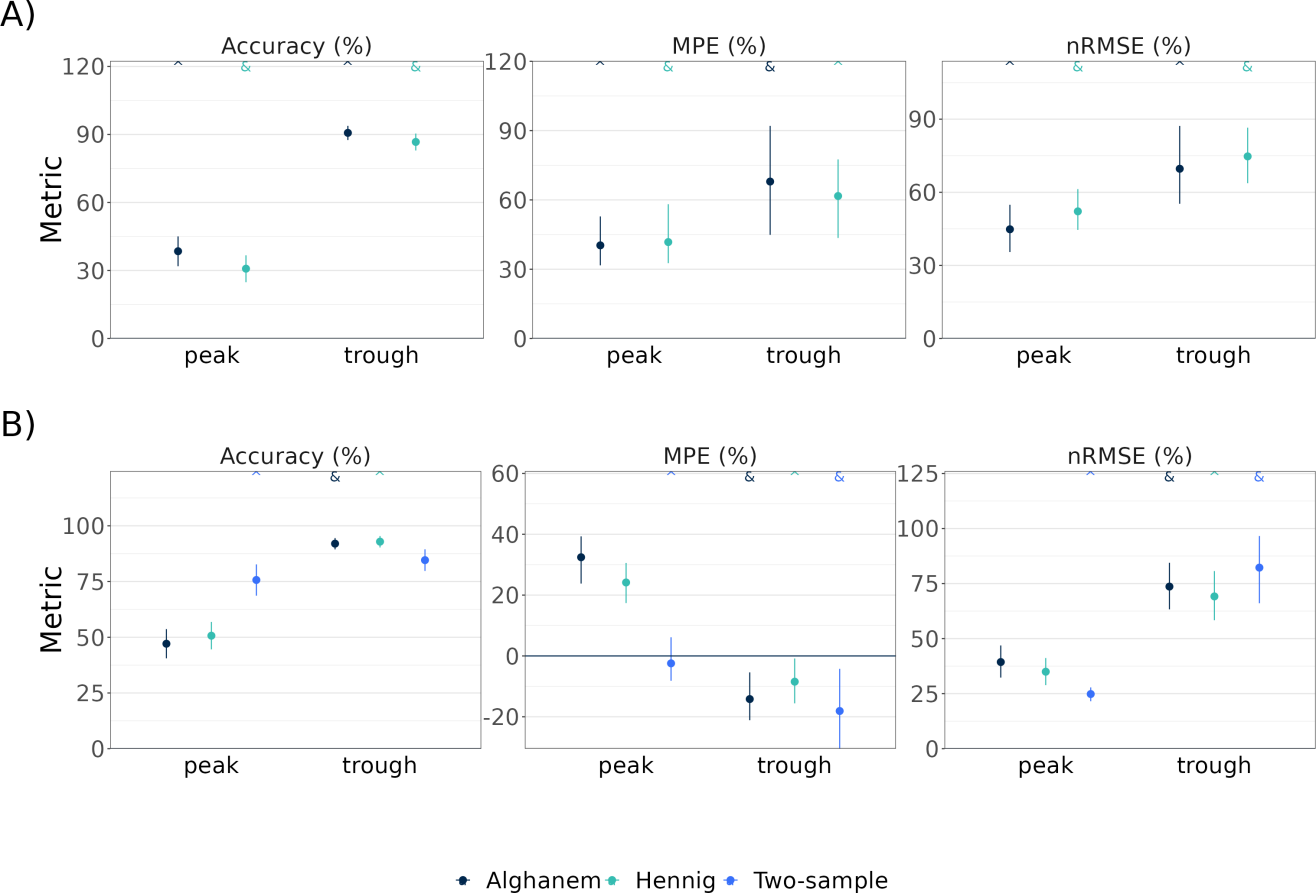
(**A**) Accuracy, mean percent error (MPE), and normalized root mean square error (nRMSE) of *a priori* predictions for the popPK models (*N* = 70 treatment courses). (**B**) Predictive performance metrics for *a posteriori* predictions (*N* = 47 for popPK models; *N* = 30 for LLR). The symbol ^ indicates the best model; the symbol & indicates models that are not statistically distinguishable from the best model.

Bayesian estimation methods could be used on all treatment courses, whereas 7 treatment courses did not have samples correctly timed for the two-sample LLR method, 7 did not have sufficient samples to evaluate predictions, and 14 had an *a priori* sample below LOQ which did not allow LLR use. The LLR method showed significantly higher accuracy (75.7% vs 50.7% Hennig and 47.1% Alghanem), lower MPE (−2.4% vs 24.2% Hennig and 32.4% Alghanem), and nRMSE (24.8% vs 34.9% Hennig and 39.4% Alghanem) in peak predictions. Bayesian estimation showed similar accuracy (92.9% Hennig and 92.0% Alghanem vs 84.6% LLR), lower MPE (−8.5% Hennig and −14.2% Alghanem vs −18.1% LLR), and nRMSE (69.2% Hennig and 73.6% Alghanem vs 82.2% LLR) in trough predictions (Fig. 2; see Fig. S3 including BLOQ concentrations). Comparing the 42 treatment courses where both methods could be evaluated, the same qualitative results hold true (Fig. S4).

Because the underestimation of peaks suggested misspecification between the typical PK parameters described by the models and this patient population, PK model performance was also evaluated with flattened priors. Prior weights were varied from 1.0 (i.e., standard MAP estimation) to 0.1, increasing the influence of patient TDM sample measurements during PK parameter estimation (15). Figure S5 shows that reducing model prior weights to 0.1 significantly improved the accuracy (up to 71.0% for Hennig and 78.3% for Alghanem vs 75.7% for LLR, indicated by the dashed line) and reduced the error (nRMSE 29.1% Hennig, 29.9% Alghanem, 24.8% LLR) and bias (MPE 7.8% for both models, −2.4% LLR) of the popPK models for peak predictions. Trough prediction performance matches or exceeds the two-sample LLR method with this flattened prior, with median accuracy of 92.9% Hennig, 92.0% Alghanem, and 84.6% LLR, median MPE of −4.2% Hennig, −17.8% Alghanem, −18.1% LLR, and median nRMSE of 71.7% Hennig, 68.8% Alghanem, and 82.2% LLR. These results suggest existing PK models are not well-suited for this CF patient population, but PK models with flattened priors can match LLR methods in performance.

### AUC estimation

**Fig 3:**
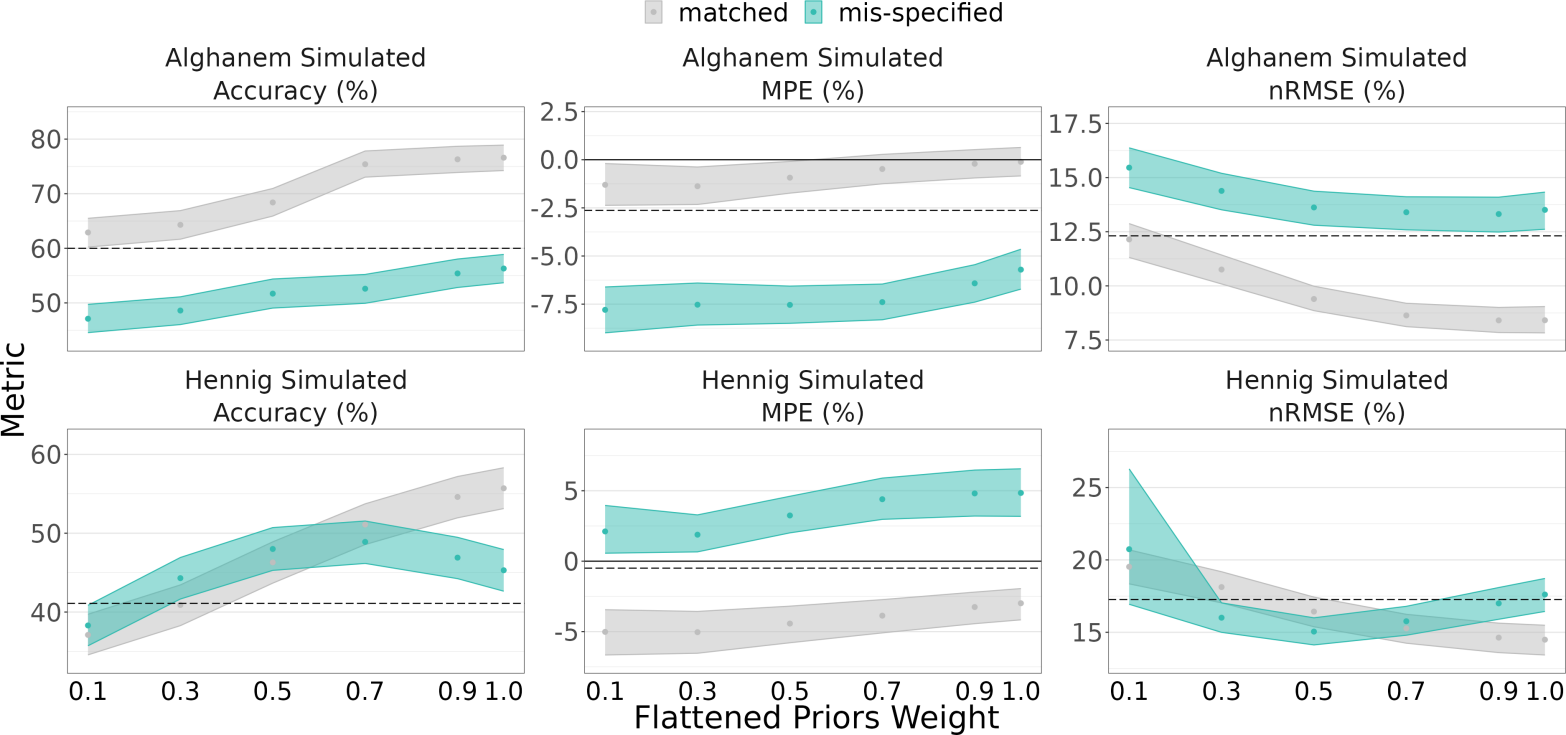
Accuracy, mean percent error (MPE), and normalized root mean square error (nRMSE) of tobramycin area under the curve (AUC) estimation with different flattened prior weights. Simulated data are from Alghanem (top row) and Hennig (bottom row) models. Matched (gray) indicates that the estimation and simulation models are the same; mis-specified (teal) is the opposite. Ribbons indicate 5th–95th percentiles of 1,000 simulated bootstraps of the data. Dashed line represents the LLR method using samples at 1 and 10 h.

When the Alghanem model was used to simulate, the LLR method estimated AUC with accuracy 60.0%, MPE −2.6%, and nRMSE 12.3%. MAP Bayesian estimation achieved 56.3%, −5.7%, and 13.5% using mis-specified popPK models with regular priors and 76.6%, −0.1%, and 8.4% for Bayesian estimation using matched popPK models with regular priors (Fig. 3). Using flattened priors with a weight of 0.5 decreased popPK model estimation performance (accuracy 51.7%, MPE −7.5%, and nRMSE 13.6%).

When the Hennig model was used to simulate, LLR estimated AUC with accuracy 41.1%, MPE −0.5%, and nRMSE 17.3%. Using the mis-specified Alghanem model with regular priors achieved 45.3%, 4.8%, and 17.6%, while flattened priors with a weight of 0.5 improved estimates to 48.0%, 3.2%, and 15.0% using a prior weight of 0.5 (Fig. 3). Matched models with full weights improved performance to 55.7%, −3.0%, and 14.5%. However, while flattened priors improved estimation using mis-specified models under Hennig simulated data, matched models (Fig. 3, gray bands) showed worsened estimation.

### Reduced sampling schemes

**Fig 4:**
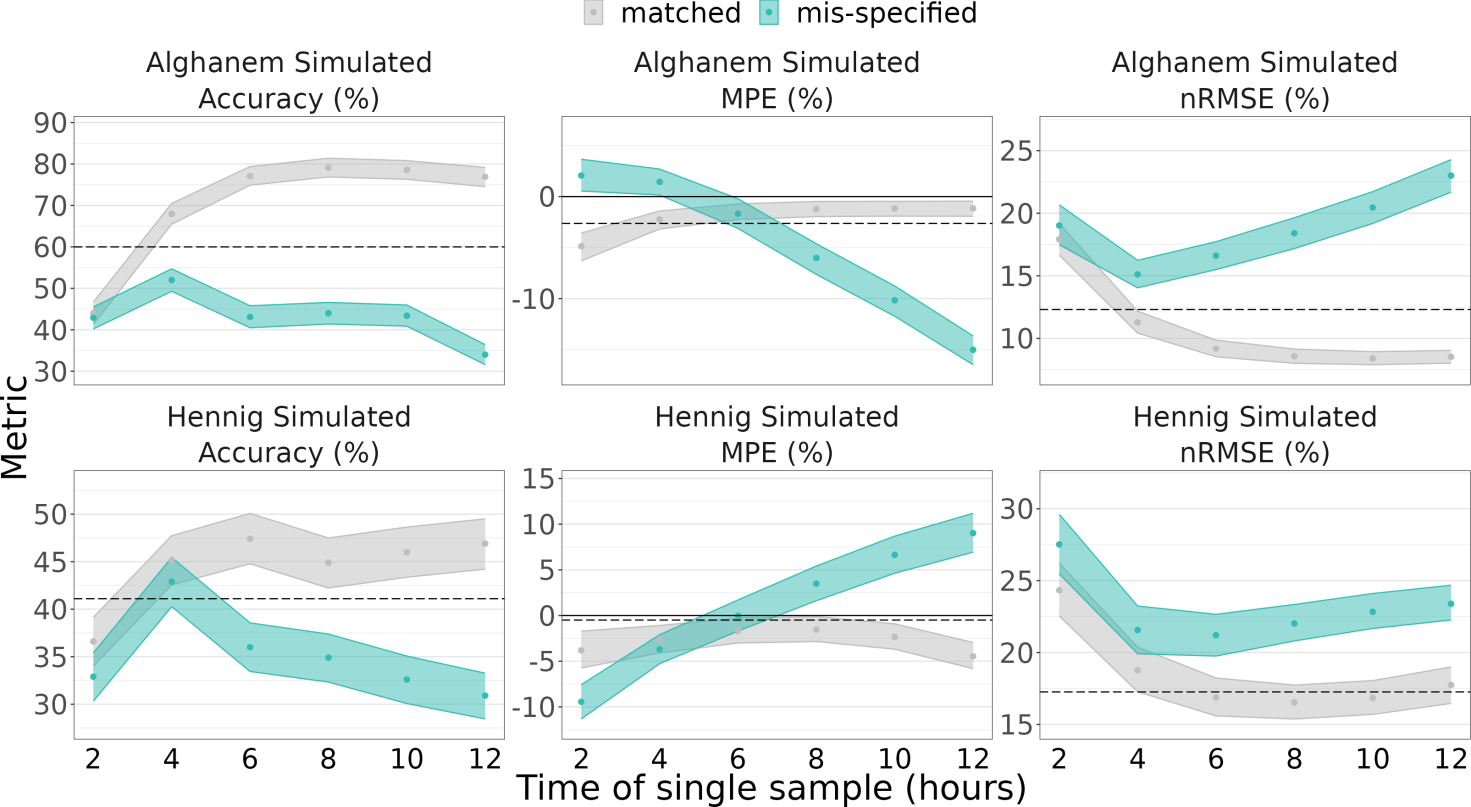
Accuracy, mean percent error (MPE), and normalized root mean square error (nRMSE) of tobramycin area under the curve (AUC) estimation under different sample timings and prior weight of 0.5. Simulated data are from Alghanem (top row) and Hennig (bottom row) models. Matched (gray) indicates that the estimation and simulation models are the same; mis-specified (teal) is the opposite. Ribbons indicate 5th–95th percentiles of 1,000 simulated bootstraps of the data. Dashed line represents the LLR method using samples at 1 and 10 h.

Bayesian estimation using a single sample taken at 4 h post-dose with a prior weight of 0.5 is on par or only slightly worse than LLR with two samples (Fig. 4; see Fig. S6 for prior weight of 1.0). With Hennig simulated data, the mis-specified Alghanem model with a prior weight of 0.5 approaches LLR performance MPE (−3.7% vs −0.5%) and nRMSE (21.6% vs 17.3%) and is better on accuracy (42.9% vs 41.1%). With Alghanem simulated data, the mis-specified Hennig model with a prior weight of 0.5 approaches LLR performance on nRMSE (15.1% vs 12.3%) and accuracy (51.7% vs 60.0%) but is better on MPE (1.4% vs −2.6%).

## DISCUSSION

Bayesian MIPD allows dose personalization earlier in therapy and with fewer samples compared to LLR, possibly reducing nursing demands and sample costs. However, MIPD relies on a sufficiently predictive PK model. Here, we evaluated popPK model and LLR predictions using only information known at the time to make the next prediction, which accurately reflects clinical care. The two models investigated here showed considerable bias in describing peak concentrations and thus estimating AUC compared to the data-driven LLR approach. However, when combined with flattened priors, which gives the models more flexibility to adapt to the data, Bayesian estimation with one sample more closely matched the performance of two-sample LLR in AUC estimation.

When the popPK model used for AUC estimation matched the model used for simulation (best case), the model-based Bayesian estimation method outperformed the LLR method, highlighting the importance of a well-specified model for prediction. When the model used for estimation was mis-specified compared to the one used for simulation (worst case), error and accuracy were improved when using flattened priors for the Alghanem model, which has fewer variability terms and lower residual error than the Hennig model. The popPK models predicted peaks poorly, which suggests a poorly estimated volume of distribution, since peaks are highly correlated with this parameter. This may be because these models were developed more than a decade ago on European (26) and Australian (25) patients, which differ from this study’s U.S.-based patient population. These models were also developed on densely sampled patients who received multiple smaller doses vs the higher daily dose now used in clinical practice.

Our results show that these popPK models are mis-specified for this patient population and improving these models by refitting or developing a new model is needed. In other drugs like vancomycin (27) and gentamicin (28), continuous learning, in which data collected from routine clinical care are used to update model parameters, has been shown to improve prediction accuracy. This process could be used in the CF patient population to improve model performance. Selectively flattening model priors when model predictions do not align well with the data has also been shown to improve prediction performance in vancomycin (15) and is another tool to enhance Bayesian estimation performance.

A single-concentration Bayesian estimation approach could provide cost savings and efficiency in workflows for the clinical team. When using a mis-specified model to estimate AUCs, a single sample at 4 h performs comparably to LLR and offers similar accuracy to Bayesian estimation with two samples at 1 and 10 h, with slightly less bias but higher nRMSE. This indicates that one sample at 4 h can serve most patients but may predict some patients with larger error.

Optimal sampling theory shows that the most informative time for estimating clearance is 1.44 half-lives of the drug, which is about 4.3 h for tobramycin (12). While a single mid-interval sample may be less convenient for hospital staff due to delayed collection after dose, its reduced cost, along with the use of MIPD software for clinically relevant metrics like peak, trough, and AUC values, more than outweighs the downsides of this approach. Previous work has also shown that a single sample 2–6 h after dose provides comparable AUC estimation to two samples at 2 and 6–8 h (29), highlighting that well-timed single samples can reduce operational challenges for hospital staff without compromising accurate PK estimation.

Forty of 70 treatment courses could not be evaluated using the LLR method due to inadequate sampling, sample collection timing issues, or samples below the LOQ, highlighting a significant limitation of this approach. LLR ignores all known population data about tobramycin and discards all previous data about a patient. LLR also assumes a linear infusion phase, leading to biases during infusion and potential mis-specification of peaks (30). This, combined with the assumption of one compartment kinetics, means that LLR is theoretically inferior on predictive performance compared to Bayesian estimation using two-compartment popPK models. Our results here show parity of Bayesian estimation to the LLR method but refitted popPK models using data more suited to modern tobramycin dosing should perform better.

This work is limited by data from a single site of adult patients with CF. Further work with more patients with and without CF at more sites would better generalize these results. Another limitation is that both models showed some misspecification, which provides some uncertainty in simulations of AUC estimations based on these models. However, simulations showed agreement with results seen in vancomycin models with respect to sample timing and sample reduction.

### Conclusion

Bayesian MIPD tools, particularly those with electronic health record (EHR) integrations, are becoming more accessible at the bedside to predict individual drug exposures. Here, we found that Bayesian estimation predicted tobramycin troughs with similar performance to the two-sample LLR method and peaks with worse error and bias. Flattening model priors, where the priors were de-emphasized relative to the data, resulted in improved Bayesian estimation, especially for tobramycin peaks. AUC estimation using Bayesian methods with flattened priors matched or bettered predictive performance in some cases compared to the two-sample LLR method on simulated data. Using one sample 4 h post-dose with flattened priors showed that single-concentration monitoring of tobramycin in adult patients with CF is feasible when using Bayesian methods for predicting AUCs. However, further work is needed to improve model predictiveness in CF patients.

### Key points

- In a real-world data set of 70 treatment courses, the current standard of two-sample log-linear regression predicted tobramycin peak concentrations better than Bayesian methods, but Bayesian estimation predicted better on non-peak concentrations.
- In a simulation study of tobramycin area under the curve estimation, using a single tobramycin sample at 4 h post-dose with Bayesian estimation was comparable to the clinical standard of two-sample log-linear regression.
- Using flattened priors, where model priors were given less weight in Bayesian estimation, also improved prediction performance when using published population pharmacokinetic models.

## Data Availability

Due to its proprietary nature, supporting data cannot be made available.

## SUPPLEMENTAL MATERIAL

Supplemental material aac.01040-24-s0001.docx Figures S1 to S6 and model files.

ASM does not own the copyrights to Supplemental Material that may be linked to, or accessed through, an article. The authors have granted ASM a non-exclusive, world-wide license to publish the Supplemental Material files. Please contact the corresponding author directly for reuse.
