# Supplementary material for: Comparing two-sample log-linear exposure estimation with Bayesian model-informed precision dosing of tobramycin in adult patients with cystic fibrosis
Source: Antimicrob Agents Chemother. 2025 Jan 10;69(2):e01040-24. doi: 10.1128/aac.01040-24 (PMC11823644; doi:10.1128/aac.01040-24)
Supplement: Supplemental material — Figures S1 to S6 and model files. [file aac.01040-24-s0001.docx]

# Supplemental Information


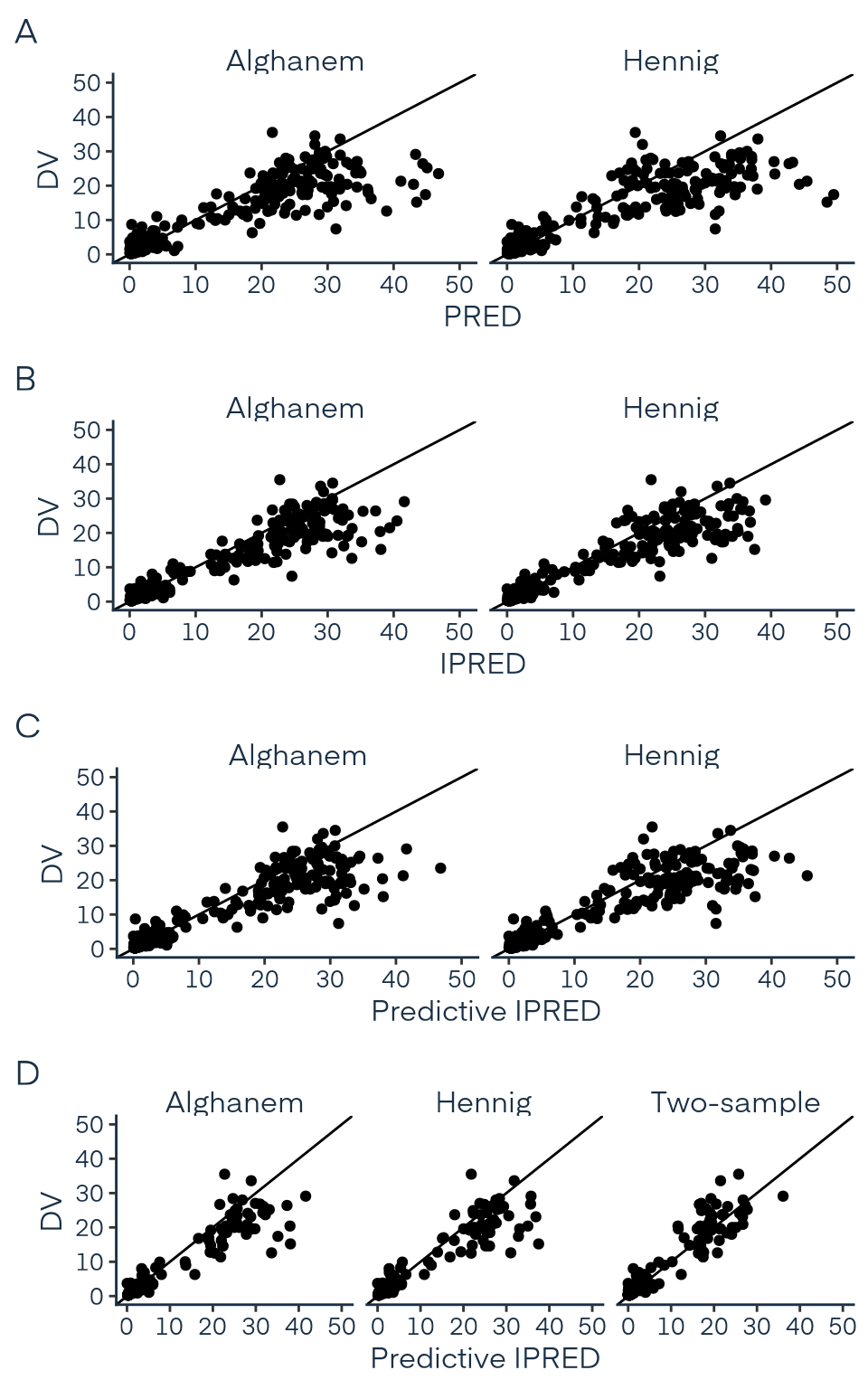


**Figure S1.** (A) DV-PRED, (B) DV-IPRED, (C) DV-predictive IPRED plots for the Alghanem and Hennig population pharmacokinetic models. (D) DV-predictive IPRED plot for tobramycin concentrations that can be predicted using the two-sample method.


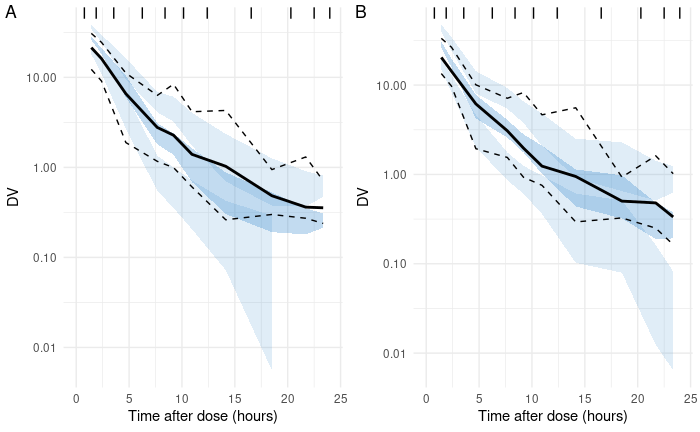


**Figure S2.** Prediction-corrected visual predictive check (pcVPC) plots for (A) Hennig and (B) Alghanem models. DV is the dependent variable, which is tobramycin concentration. Black dashed lines represent the 10^th^ and 90^th^ percentiles of the observed tobramycin concentration; black solid line represents the median. Shaded ribbons denote the 95^th^ confidence intervals for the simulated 10^th^, 50^th^, and 90^th^ percentiles of tobramycin concentrations.


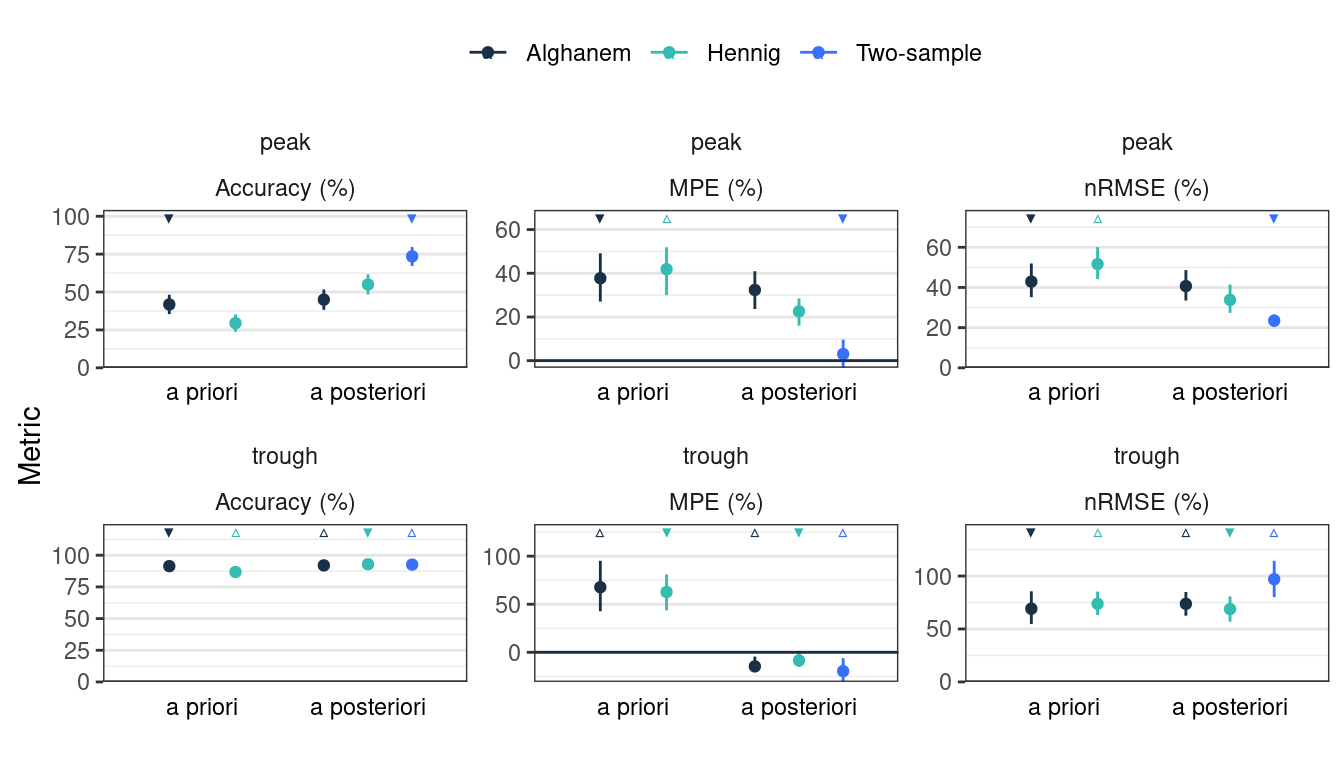


**Figure S3.** Accuracy, mean percent error (MPE), and root mean square error (RMSE) of predictions for the popPK models and two-sample LLR without discarding below limit of quantification samples. Solid triangles indicate the best model; hollow triangles indicate models that are not statistically distinguishable from the best model. *A priori* predictions are predictions made before any tobramycin level is drawn (N = 70 treatment courses for popPK models; N = 0 for two-sample); *a posteriori* predictions are made after fitting with tobramycin level(s) (N = 47 for popPK models; N = 42 for two-sample method).


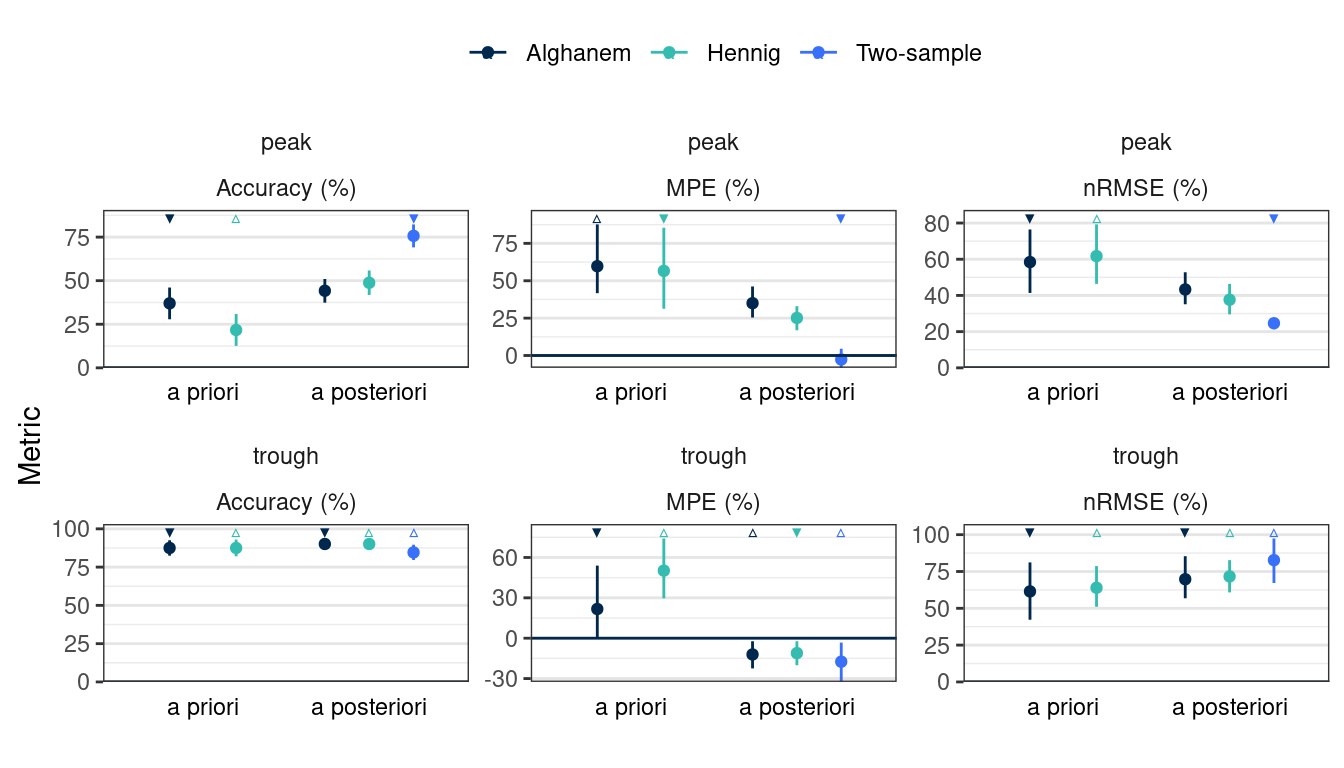


**Figure S4.** Accuracy, mean percent error (MPE), and root mean square error (RMSE) of peak and trough predictions for the Alghanem and Hennig models, and the two-sample method in adult cystic fibrosis patients dosed with tobramycin where the two-sample method could be used. Solid triangles indicate the best model; hollow triangles indicate models that are not statistically distinguishable from the best model. *A priori* predictions are predictions made before any tobramycin level is drawn; *a posteriori* predictions are made with tobramycin level(s) as needed by the estimation method.


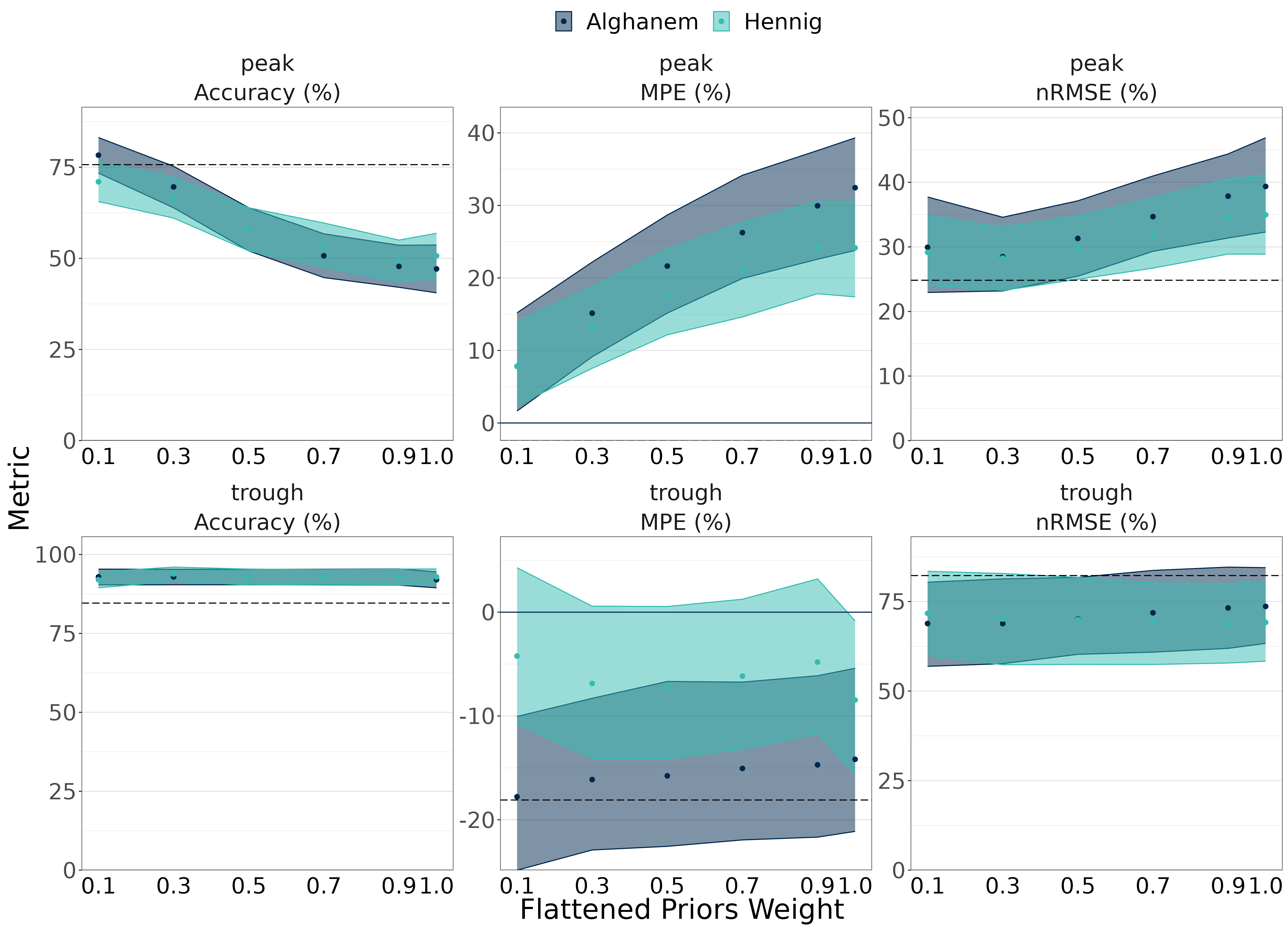


**Figure S5.** Accuracy, mean percent error (MPE), and root mean square error (RMSE) of *a posteriori* peak and trough predictions for the Alghanem and Hennig models using flattened priors and samples at 1 and 10 hours. Tobramycin concentration predictions were made with flattened priors weights of 0.1, 0.3, 0.5, 0.7, 0.9, and 1.0, where lower weights represent more estimation weight on the tobramycin sample. Ribbons indicate 5th and 95th percentiles of 1000 simulated bootstraps of the data. Dashed line represents the standard of care two-sample method.

**
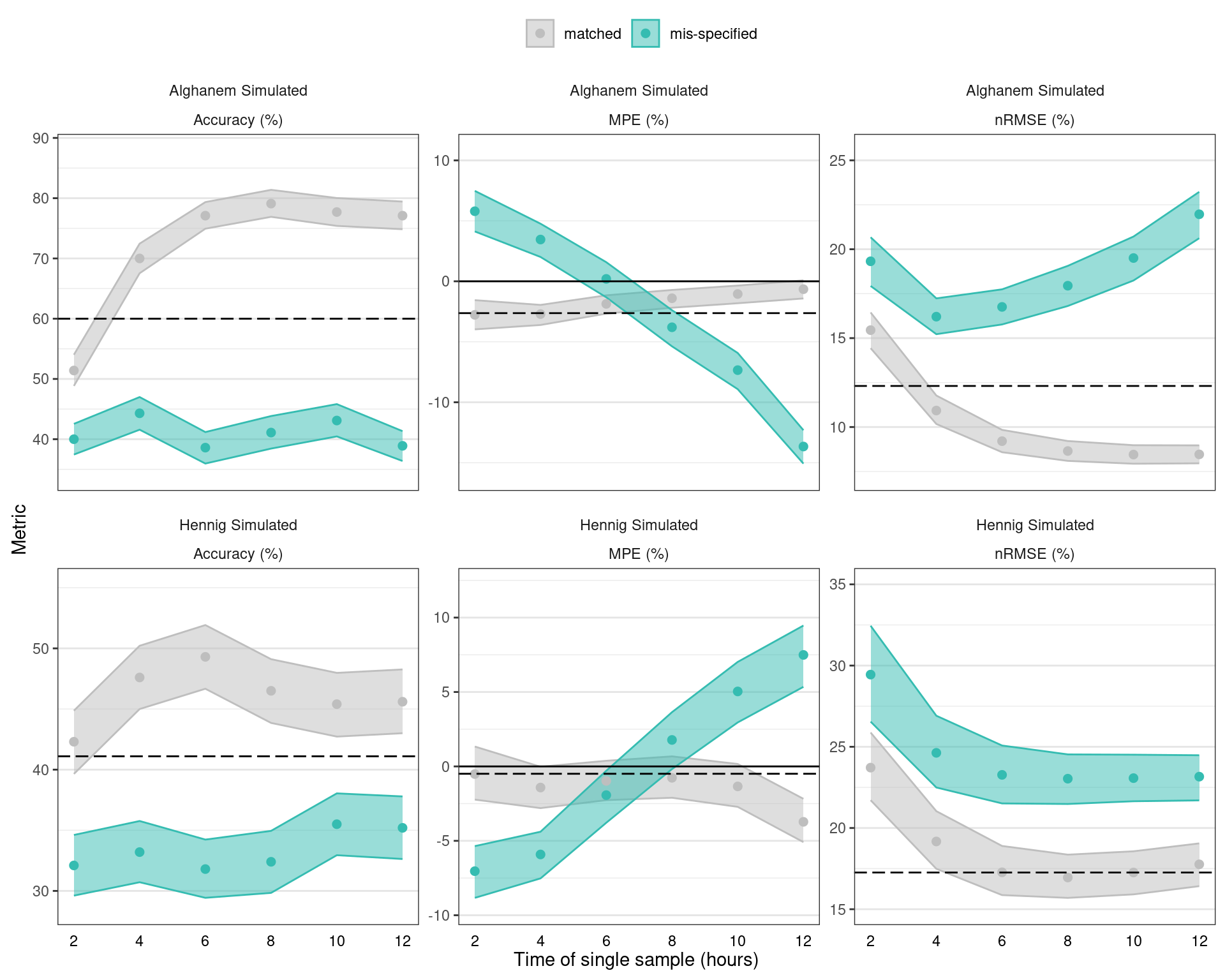
 Figure S6.** Accuracy, mean percent error (MPE), and normalized root mean square error (nRMSE) of tobramycin area under the curve (AUC) estimation under different single sample timings at a flattened prior weight of 1. Simulated data are from Alghanem (top row) and Hennig (bottom row) models. Matched (gray) indicates that the estimation and simulation models are the same; mis-specified (teal) is the opposite. Ribbons indicate 5^th^-95^th^ percentiles of 1000 simulated bootstraps of the data. Dashed line represents the LLR method using samples at 1 and 10 hours.

## Hennig model with flattened priors weight of 0.5 in NONMEM

$PROBLEM dummy

$INPUT ID OCC TIME EVID MDV DV AMT LENGTH RATE SEX AGE CR FFM WT HT CRCL CL_HEMO

$DATA nm_data.dat

$SUBROUTINE ADVAN3 TRANS4

$PK

BP=18

IF(AGE.LE.BP) FAGE =THETA(6)*(AGE - 18)

IF(AGE.GT.BP) FAGE =THETA(7)*(AGE - 18)

; expected Serum creatinine for age and sex:

MEANSCR = 1

IF(AGE <= 15) THEN

MEANSCR = -2.33730 - (12.91367 * log(AGE)) + 23.93581 * sqrt(AGE)

ENDIF

IF(AGE > 15 .AND. AGE < 18) THEN

IF(SEX == 0) THEN

MEANSCR = 9.5471 * AGE - 87.847

ELSE

MEANSCR = 4.7137 * AGE - 15.347

ENDIF

ENDIF

IF(AGE >= 18) THEN

IF(SEX == 0) THEN

MEANSCR = 84

ELSE

MEANSCR = 69.5

ENDIF

ENDIF

FSCR=(MEANSCR/(CR*88.4))**THETA(8) ; MEANSCR is mean for age and sex.

; SEX = 1 is female

TVCL = THETA(1) * (FFM/70)**(THETA(5)) * (1+FAGE) * FSCR * (THETA(9)) ** (1 - SEX)

TVV1 = THETA(2) * (FFM/70) * (THETA(10)) ** (1 - SEX)

TVQ = THETA(3) * (FFM/70)**(THETA(5))

TVV2 = THETA(4) * (FFM/70)

; IOV

BOVCL = 0

IF(TIME.LE.24) BOVCL = ETA(5)

IF(TIME.GT.24) BOVCL = ETA(6)

IF(TIME.GT.48) BOVCL = ETA(7)

CL = TVCL * EXP(ETA(1)+BOVCL)

V1 = TVV1 * EXP(ETA(2))

Q = TVQ * EXP(ETA(3))

V2 = TVV2 * EXP(ETA(4))

S1 = V1

$ERROR

IPRED = F

PROP = 0.204

ADD = 0.001

W = SQRT(PROP**2 * IPRED**2 + ADD **2)

Y = IPRED + W * EPS(1)

$THETA

8.1 FIX ; CL female

20.1 FIX ; V1 female

1.5 FIX ; Q

10.0 FIX ; V2

0.952 FIX ; EXP FFM on CL

-0.021 FIX ; AGE on CL <18

-0.010 FIX ; AGE on CL >18

0.222 FIX ; EXP SCR on CL

(0, 1.160) FIX ; SEX on CL

(0, 1.249) FIX ; SEX on V1

$OMEGA BLOCK(3)

0.26832

0.10360 0.09240

0.30788 0.12072 0.69880

$OMEGA 1.36892

$OMEGA BLOCK(1) 0.01613 ; BOVCL1

$OMEGA BLOCK(1) SAME

$OMEGA BLOCK(1) SAME

$SIGMA

1 FIX

$EST METHOD=1 MAXEVAL=0 PRINT=5

## Alghanem model with flattened priors weight of 0.5 in NONMEM

$PROBLEM dummy

$INPUT ID OCC TIME EVID MDV DV AMT LENGTH RATE SEX AGE CR FFM WT HT CRCL CL_HEMO

$DATA nm_data.dat

$SUBROUTINES ADVAN3 TRANS4

$PK

IF(NEWIND.NE.2) THEN

TAD = 0

TLASTDOSE = 0

ENDIF

IF(EVID.EQ.1) TLASTDOSE = TIME

TAD = TIME - TLASTDOSE

TVCL = THETA(1) * HT + THETA(2) * (CRCL - 92)

TVV1 = THETA(3) * (1 + THETA(4) * (HT - 163))

TVV2 = THETA(5)

TVQ = THETA(6)

; IOV

IF(TIME.LE.24) BOVCL = ETA(3)

IF(TIME.GT.24) BOVCL = ETA(4)

IF(TIME.GT.48) BOVCL = ETA(5)

IF(TIME.GT.72) BOVCL = ETA(6)

CL = TVCL * EXP(ETA(1)+BOVCL)

V1 = TVV1 * EXP(ETA(2))

Q = TVQ

V2 = TVV2

S1 = V1

$ERROR

IPRED = F

PROP = 0.148

ADD = 0.086

W = SQRT(PROP**2 * IPRED**2 + ADD **2)

$THETA

0.0285 ; HT on CL

0.0114 ; renal on CL

13.3 ; TVV1

0.0113 ; HT on V1

6.62 ; V2

0.452 ; Q

$OMEGA

0.129600

0.053824

$OMEGA BLOCK(1) 0.012996 ; BOVCL1

$OMEGA BLOCK(1) SAME

$OMEGA BLOCK(1) SAME

$OMEGA BLOCK(1) SAME

$SIGMA

1

$ESTIMATION MAXEVAL=0 METHOD=1
